# P62 Penicillin allergy de-labelling as part of medication history taking—a pharmacy technician-led improvement project

**DOI:** 10.1093/jacamr/dlag102.068

**Published:** 2026-06-26

**Authors:** Georgina Hutchinson, Zara Tariq, Sarah Denman

**Affiliations:** Leeds Teaching Hospitals NHS Trust, Leeds, England; Leeds Teaching Hospitals NHS Trust, Leeds, England; Leeds Teaching Hospitals NHS Trust, Leeds, England

## Abstract

**Background:**

Around 5.6% of the population carry a penicillin allergy label, affecting an estimated 2.7 million people in the UK, including about 46 000 in Leeds (West et al., 2019). Recent evidence shows these labels are still not routinely reassessed, contributing to antimicrobial resistance (Krishna et al., 2025). At LTHT and internationally, incorrect labels are typically removed by taking an accurate history, identifying cases incompatible with true allergy, and performing a direct oral penicillin challenge (Bestwick et al., 2025; Leeds Teaching Hospitals, 2024). Thorough assessment is essential to avoid unnecessary avoidance of first-line antibiotics while ensuring patient safety. Although LTHT guidelines outline the questions needed for a full allergy history, many pharmacy technicians report that these are too clinical, leading to brief and incomplete documentation. As a result, patients may retain inaccurate allergy labels, limiting access to appropriate first-line antimicrobials.

**Objectives:**

To highlight patients for de-labelling by confirming medication histories and identifying which patients had true allergies versus those experiencing side effects to penicillins.

**Methods:**

A two-week baseline audit was conducted on an acute admissions ward to identify patients with a documented penicillin allergy. Patients aged 18–75 were included. Allergy information was gathered from the patient or carer, GP records, and electronic medical notes. Using LTHT guidelines, I determined which patients had a true penicillin allergy and which were experiencing side effects and could be considered for de-labelling. A two-week intervention audit was then completed on the same ward using the same criteria, with the added step of placing a clinical note in the patient’s record to flag those requiring review for de-labelling. The pharmacist was informed, and the note was visible to the wider MDT, who could either carry out the de-labelling process or add the recommendation to the discharge advice for the GP to action. A follow-up review confirmed whether the inpatient team or GP had acted on the de-labelling recommendation.

**Results:**

In the baseline audit, 27 patients were reviewed. Of these, 21 were suitable for a penicillin challenge and potential de-labelling. 20/27 patients had a documented penicillin allergy; 15 described reactions consistent with side effects, while 5 had true allergies. 14 patients received antimicrobials during admission, including 2 who were prescribed amoxicillin—1 of whom had amoxicillin listed as an allergy. None of the patients had their penicillin allergy status reviewed by a clinician, pharmacist, or pharmacy technician. During the intervention audit, 23 patients had a documented penicillin allergy. 12/23 had an accurate allergy recorded, ten had side effects documented, and one had an unknown reaction. 7/10 patients with side effects met the criteria for de-labelling. 5 of these required antimicrobials; according to LTHT guidelines, 4 could have been prescribed a penicillin for their infection. All 7 had a clinical note added to their record and were followed for two weeks to see whether the MDT or GP acted on the recommendation. 1 patient died during admission, and the remaining 6 were discharged with no action taken and no documentation added to their eDAN. Consequently, none of the inaccurate penicillin allergy labels were challenged or removed.

**Conclusions:**

The two-week follow-up period showed that none of the highlighted patients were de-labelled by either the inpatient or community teams. This reinforces the need for greater MDT involvement in supporting penicillin de-labelling when suitable patients are identified. Improving the accuracy of allergy documentation—including confirming the allergy and the reaction described—remains essential, and pharmacy technicians have a key role in strengthening this process during the medication history stage of admission.

